# Oral Hygiene Considerations in Adult Patients with Leukemia during a Cycle of Chemotherapy

**DOI:** 10.3390/ijerph19010479

**Published:** 2022-01-02

**Authors:** Maja Ptasiewicz, Paweł Maksymiuk, Renata Chałas

**Affiliations:** Department of Oral Medicine, Medical University of Lublin, ul. Chodźki 6, 20-093 Lublin, Poland; maja.ptasiewicz@umlub.pl (M.P.); renata.chalas@umlub.pl (R.C.)

**Keywords:** leukemia, periodontal status, oral hygiene, chemotherapy, oral health

## Abstract

The oral cavity is the place where the first symptoms of systemic diseases may appear. Leukemia is the malignancy of the hematopoietic system in which abnormal leukocytes are produced in the bone marrow and these cells spread to the peripheral blood. It is classified clinically on the basis of the duration and nature of the disease (acute or chronic), the type of cell involved (myeloid, lymphoid, or monocytes), and a rise in the number of abnormal cells in the blood. The study aimed to assess and compare the oral hygiene and periodontium status based on the indices in leukemic patients before and after one cycle of chemotherapy and whether the therapy had an impact on the change of these parameters. Dental indices used in clinical diagnostics were calculated: API (approximal plaque index), SBI (sulcus bleeding index), and CPI (community periodontal index). The research project was conducted at the Clinic of Hematooncology and Bone Marrow Transplantation of the Independent Public Clinical Hospital No. 1 in Lublin. The target population consisted of 102 adults with leukemia who were over 18 years of age. The time since diagnosis of the disease ranged from 1 to 10 years. The data were evaluated in the Statistica 12 software with the respective tests. In the majority of patients, both before and after chemotherapy, improper oral hygiene and severe generalized periodontitis were confirmed. The cycle of chemotherapy that was used did not correlate with the change of patients’ oral hygiene and periodontium state. Unsatisfactory oral hygiene and periodontal health has to be addressed with urgent dental treatment to avoid systemic complications in leukemic patients.

## 1. Introduction

The oral cavity is the place where the first symptoms of systemic diseases may appear. Often, signs of general diseases are seen in the mouth even before the underlying disorder is diagnosed, and they can expedite diagnosis and provide a chance for prompt treatment. In approximately 20% of patients, the disease is detected on routine testing with a peripheral blood count showing significant abnormalities [1]. Leukemia is a hematopoietic malignancy in which abnormal leukocytes form in the bone marrow and spread to the peripheral blood. The blast cells replace normal cells in the bone marrow and accumulate in other organs and tissues [2]. Clinical classification is based on the duration and nature of the disease (acute or chronic), the type of cells involved (myeloid, lymphoid, or monocytes), and a rise in the number of abnormal cells in the blood [3]. The neoplastic process may spread to the lymph nodes, liver, and spleen, which is mainly the case for acute and chronic lymphoid leukemia. General symptoms, such as fever, weight loss, muscle and joint pain, and epistaxis, may occur during the course of leukemia, as well as during treatment. Some patients have general symptoms associated with anemia or thrombocytopenia, hemorrhagic diathesis, granulocytopenia, and immune disorders. Oral problems associated with this disease and its treatment may include petechiae, spontaneous bleeding and pain, mucosal ulceration, gingival hyperplasia with or without necrosis, and mucosal pallor. In some cases, dysfunction of salivary glands and temporomandibular joint is observed. Neurotoxicity, mucosal fibrosis, radiation osteonecrosis, or soft tissue necrosis may occur after the treatment ends. Patients have impaired healing due to reduced anti-inflammatory defenses and are more susceptible to viral opportunistic infections, secondary bacterial infections, or candidiasis [4]. Opportunistic infections can be caused by potential foci of infection in the oral cavity, as well as by dental plaque deposits in which many bacterial species are present.

Biofilm accumulation and poor oral hygiene are known major risk factors for gingivitis or periodontitis, especially in patients with systemic diseases, including leukemia. Epidemiological studies show that plaque-induced gingivitis, which is a reversible form of inflammation, is most commonly observed in adolescents [5]. Gingival bleeding may precede the diagnosis of the hematological disease, and it is the first symptom in the oral mucosa in 17.7 and 4.4% of patients with acute and chronic leukemia, respectively.

Oral biofilm development and maturation have been studied in vitro and in vivo, and the microbial succession of species in plaque is well described. For example, early colonizing species are usually facultative anaerobes using mainly salivary mucins or other glycoproteins [6]. During biofilm growth and maturation, there is a gradual loss of oxygen, and absolute proteolytic anaerobes increase in abundance. This leads to a fully mature oral biofilm profile including *Tannerella*, *Porphyromonas*, *Metanobrevibacter*, and *Desulfobulbus* [7]. Frequent plaque removal through tooth brushing and professional dental cleaning has been shown to prevent full maturation of the biofilm [8].

During a patient’s hospitalization, oral biofilm growth occurs constantly. Dental deposits, such as plaque and tartar calculus, are a source of microorganisms responsible for not only oral but also systemic complications and can lead to life-threatening bacteremia, especially in immunocompromised patients. For the prevention of general inflammation, daily effective prophylactic and hygienic measures to remove plaque are fundamental: systematic tooth brushing at least twice a day (using a toothbrush and toothpaste). It is also recommended to use additional oral hygiene accessories (dental floss or irrigators) and mouthwashes [9,10].

In patients undergoing chemotherapy, oral hygiene may be difficult due to pain associated with mucositis and general weakness. It is extremely important to make patients aware of the importance of maintaining optimal oral hygiene during treatment. Low awareness of this issue can lead to deterioration of the oral hygiene in these patients, which contributes to serious overall complications.

### Aim

The study aimed to assess and compare the oral hygiene and periodontium status based on the indices in leukemic patients before and after one cycle of chemotherapy and whether the therapy had an impact on the change of these parameters.

## 2. Materials and Methods

All procedures performed in the study were in accordance with the ethical standards of the institutional and national research committee and with the 1964 Helsinki Declaration, as well as its later amendments, or comparable ethical standards. Informed consent was obtained from all participants included in the study. The project was approved by the Bioethics Committee of the Medical University of Lublin.

### 2.1. Participant Selection

The present study was a research project conducted at the Clinic of Hematooncology and Bone Marrow Transplantation of the Independent Public Clinical Hospital No. 1 in Lublin. The target population consisted of patients with leukemia over 18 years of age. All clinical data, including laboratory test results, and information about treatment and its course were obtained from hospital records. All conducted procedures during the study were performed after obtaining informed written consent from the patient.

Patients were excluded from the study if they presented any of the following criteria: age < 18 years old, receiving stem cell transplantation or radiotherapy or suffering from any other hematological disease than leukemia, using fixed orthodontic appliances, had started palliative health care, or whose general state made dental examination impossible.

### 2.2. Clinical Assessment

The first examination, to assess oral hygiene and periodontal status, was performed before the treatment phase, i.e., before the next cycle of chemotherapy. Another examination was conducted after the completion of the chemotherapy cycle—after 7–14 days, depending on the type of leukemia and according to the treatment protocol.

Clinical oral parameters were assessed by one experienced dentist during the first and follow-up examinations after chemotherapy. The researcher was prepared and calibrated by the experienced clinician. Before the study, intraexaminer reproducibility was assessed for this examiner and proved to be adequate to perform the study. A basic dental diagnostic kit with a WHO-calibrated periodontal probe (WHO 621) was used to record the probing measurements. The probe tip had a 0.5 mm diameter ball and millimeter markings of 3.5, 8.5, and 11.5, and color markings of 3.5–5.5 mm and 8.5–11.5 mm. During oral examination, attention was paid to spontaneous gingival bleeding or on gentle probing, tooth mobility, and the presence of supragingival and subgingival dental deposits. Periodontal pockets and their depth were also assessed. The following indices—SBI, API, and CPI—were used to assess oral hygiene and periodontal status [11].

#### 2.2.1. SBI Index Assessment

SBI—(sulcus bleeding index) a modified sulcus bleeding index. The presence or absence of bleeding on probing the gingival pocket is taken into account. The index is calculated according to the formula:

$$\mathrm{SBI}=\frac{\mathrm{The}\;\mathrm{number}\;\mathrm{of}\;\mathrm{bleeding}\;\mathrm{gingival}\;\mathrm{units}}{\mathrm{The}\;\mathrm{number}\;\mathrm{of}\;\mathrm{all}\;\mathrm{examined}\;\mathrm{gingival}\;\mathrm{units}}\times 100\%$$

The periodontium state is assessed basing on the ranges of index value:

SBI 100–50%—severe generalized periodontitis;

SBI 49–20%—moderate periodontitis requiring intensive treatment;

SBI 19–10%—mild periodontitis, the state requiring improvement;

SBI < 10%—clinically healthy periodontium, the state of dentition was stabilized.

#### 2.2.2. API Index Assessment

API—(approximal plaque index) approximal plaque index allows simplified clinical assessment of the level of oral hygiene. The presence of interdental deposits is assessed. In the first and third quadrant, the examination is carried out from the side of oral cavity proper and, in the second and fourth quadrant, from the oral vestibule. The API index is calculated as follows:

$$\mathrm{API}=\frac{\mathrm{Amount}\;\mathrm{of}\;\mathrm{interdental}\;\mathrm{spaces}\;\mathrm{with}\;\mathrm{plaque}}{\mathrm{The}\;\mathrm{sum}\;\mathrm{of}\;\mathrm{all}\;\mathrm{assessed}\;\mathrm{interdental}\;\mathrm{spaces}}\times 100\%$$

The presence or lack of dental plaque is assessed, and the index value is expressed as a percentage:

API 100–70%—improper oral care;

API 69–40%—mediocre hygiene, improvement is necessary;

API 39–25%—quite good oral care, especially when the index is close to 25%. With a value below 30%, it can be assumed that there are proper conditions for the protection against dental caries and periodontal diseases;

API < 25%—optimum oral hygiene.

The expression of effective hygiene procedures is reaching the API level of minimum 35%.

#### 2.2.3. CPITN Index Assessment

CPI—(community periodontal index) index of the state of periodontium (according to the World Health Organization, WHO), it includes the code of morbid symptoms (0–4). The corresponding treatment needs (TN) I–III are added according to CPI values.

When examining CPITN index, the patient’s dentition should be divided into groups (sextants) involving molars and premolars on both sides of dental arches (lateral groups) and the teeth from the canine to canine (anterior groups). The examination is performed with the use of calibrated periodontal probe with a round tip 0.5mm in diameter. In adults, all teeth are examined, and the highest value of each sextant is taken into account. If, in the investigated group, there are not at least two fully functioning teeth, such a segment is not assessed, and individual teeth are included in the adjacent groups. Teeth substantially damaged by dental caries, as well as movable teeth—grade III tooth mobility, are not subject to the assessment.

CPI allows to define all treatment needs (TN) with comprehensive dental treatment planning (Table 1).

### 2.3. Statistical Analysis

The obtained study results were submitted for statistical analysis. The calculation for quantitative traits included: range of values (min., max.), arithmetic mean (M), and standard deviation (SD). Differences between the compared groups for quantitative traits were verified with tests (depending on the stated distribution): for dependent variables—Student’s *t*, Wilcoxon; for independent variables—Student’s *t*, Mann–Whitney U test. The differences in the prevalence of the analyzed traits between particular groups were tested with χ^2^ test. The study adopted a 5% risk of error; therefore, statistically significant differences were those of *p* < 0.05. All the calculations were carried out with the Statistica 12 software package (StatSoft, Tulsa, OK, USA).

## 3. Results

### 3.1. Study Group Characteristics

Fifty-one women (50.00%) and fifty-one men (50.00%) aged 22 to 72 (54.07 ± 10.33 years) participated in the study. The time since diagnosis of the disease ranged from 1 to 10 years

The patient diagnoses were as follows in Table 2.

The general division of leukemia was used for statistical analysis: acute leukemia was diagnosed in 74 patients (72.55%) and 28 patients (27.45%) had chronic leukemia.

### 3.2. Chemotherapy Cycle versus Periodontal Indices

#### 3.2.1. Sulcus Bleeding Index

Sulcus bleeding index—SBI [%] was defined in 87 patients (85.29%). In 15 patients (14.71%), due to substantial loss of teeth, it was not assessed. The mean value during the first appointment was 63.83 ± 24.76 and, during the next appointment, it was 65.25 ± 24.54. No significant difference was stated for the SBI index between the appointments (*p* = 0.5142). The minimum SBI value was 23 and was recorded in one patient (0.98%); the maximum value—100%—was found in 13 patients before and 15 after chemotherapy (12.7 and 14.7%, respectively). The analysis of the SBI index in patients showed that all individuals had inflammation within the periodontal tissues. Before the chemotherapy, in 59 subjects (57.84%), the SBI value ranged from 50 to 100%. After hematological treatment, the SBI value was in the same range of 50–100% in 63 subjects (61.76%). Moderate inflammation of the gums indicated by SBI = 20–49% was reported in 28 people before and 24 people after chemotherapy. No patients had mild gingivitis at SBI ranging from 19 to 10%, and none had an SBI index of <10%, which indicates healthy periodontium (Table 3).

#### 3.2.2. Approximal Plaque Index

The mean API value [%] was defined in 87 patients (85.29%). In 15 patients (14.71%), due to substantial loss of teeth, it was not assessed. The mean API value was 74.38 ± 23.28 and 73.48 ± 23.57, respectively, during the first and follow-up examinations. No significant differences were stated for the API index between the appointments (*p* = 0.8816). Evaluation of the API index proved that none of the examined patients had optimal oral hygiene, 51.96% of patients had inadequate oral hygiene, in 27.45% of patients the oral hygiene was average and required improvement, and in 20.59% of examined patients the oral hygiene was assessed as fairly good. Similar results were obtained by analyzing API in patients after chemotherapy. None of the patients tested had an API of < 25%, i.e., optimal oral hygiene. Inadequate oral hygiene was found in 51 patients (50.00%); average hygiene in 30 people (22.55%); and fairly good oral hygiene was reported in six patients (22.55%) (Table 4).

#### 3.2.3. Community Periodontal Index (CPI) and Treatment Needs (TN)

The CPI index was determined in 76 patients (74.51%); in the remaining 26 people (25.49%), due to many missing and not-eligible-for-examination teeth, it was not assessed. None of the patients had a healthy periodontium (code 0). In 30 patients (29.41%), gums bleeding after gentle probing were observed (code 1). There were 25 persons (24.50%) who had super- and subgingival calculus and overhanging fillings (code 2), and 17 patients (16.6%) had pathological gingival pockets with a depth of 3.5–5.5 mm (code 3). In four persons (3.92%), gingival pockets were deeper than 6 mm (code 4). The CPI index did not change after hematological treatment (Table 5).

The first category of periodontal treatment needs (TN) was stated in 30 patients (29.41%), the second one in 42 patients (4.18%), and the third in 4 patients (3.92%) (Table 6).

The determination possibility of the CPI index was statistically significantly dependent (*p* < 0.01) on the age of the patients. The determination of CPI was possible significantly more frequently in patients under 56 years old and was determined in this group in 85.19% of patients (Table 7).

There was also a statistically significant (*p* = 0.0173) relationship between the age of the patients and the occurrence of TN categories. The first category of treatment needs was found most frequently in the respondents. Younger patients (<56 years old) constituted 73.33% of patients with TN I (Table 8).

### 3.3. Type of Leukemia Versus Periodontal Indices

There was no statistically significant correlation between API and SBI values and the type of leukemia (Table 9).

## 4. Discussion

In the case of acute leukemia, treatment depends on the type of leukemia, the patient’s age, risk factors, and the prevalence of the disease in other tissues and organs. Treatment consists of the following stages: induction, remission, consolidation, and maintenance therapy. Induction chemotherapy is still the therapy of choice for the patients with leukemia [12]. Despite multiple complications and infections caused by the immunosuppressive effects of used treatment, this therapy is often the only chance to save patients’ lives. Side effects of the therapy can be very serious and, in some cases, lead to increased mortality [13]. Microbes from the oral cavity enter the blood stream and are the major cause of bacteremia [14]. Especially invasive, surgical oral treatment in patients undergoing chemotherapy may have serious consequences [15]. Therefore, dental clearance and hygiene improvement before chemotherapy is recommended in most cases [2,10].

Proper and professional oral hygiene care and education by dental professionals are very important for patients receiving chemotherapy. However, this topic is not extensively emphasized in the professional literature concerning the adult population. There are only a few studies about oral hygiene and periodontal status in adult patients with leukemia during chemotherapy. The literature mainly presents information on the oral health of children [16,17,18,19,20,21,22].

This study aimed to evaluate the oral hygiene in patients before and after one cycle of chemotherapy, as well as the changes in periodontium status in these patients. In this study, the authors highlight the importance of good oral hygiene for the general health of adult patients undergoing chemotherapy.

In our study, we noticed no significant differences for the API index between the appointments (*p* = 0.8816). On the other hand, evaluation of the API index proved that none of the examined patients had optimal oral hygiene both before and after a cycle of chemotherapy. The majority of patients with leukemia had poor oral hygiene. Our observation is supported by other studies measuring the API index of adult leukemic patients [23,24].

This could be related to irregular brushing habits because of the difficulties, pain, and bleeding or due to lack of basic knowledge about the health effects of poor oral hygiene, resulting in an insufficient tooth-brushing technique. Dontasky et al. found that the oral health status of hospitalized patients diagnosed with leukemia is generally poor [25]. Poor oral hygiene defined by the API index and gingival bleeding assessed on the basis of the SBI index are factors of increased risk of infectious complications in hospitalized patients during chemotherapy. Improper oral hygiene is a well-known risk factor for leukemic gingival hyperplasia, periodontitis, and tissue necrosis, predisposing patients to oral pain. General symptoms, such as fever, malaise, and weakness, are all manifestations of advanced cases of leukemia. Patients with improper oral hygiene are also more likely to suffer from viral, bacterial, and fungal infections. That may complicate oncological treatment, negatively affecting patient’s health. In patients undergoing chemotherapy, odontogenic infections can develop into life-threatening sepsis [26,27]. In numerous articles, the researchers reported that the most common oral signs of leukemia occurring after diagnosis were gingival bleeding and reddening. Some authors found that patients with acute leukemia who have these symptoms tend to have a shorter survival time as compared to patients who do not show these lesions [28]. Schmalz et al., in the respective pilot study, included 39 patients suffering from acute leukemia, and a periodontal treatment need was found for 76% of participants [29]. The authors found a poor oral health condition in adult patients with acute leukemia and concluded that improved dental care was necessary for those patients. In our study, the CPI (community periodontal index) was evaluated in 76 patients (74.51% of the study group); in the remaining group (26 people, 25.49%), due to many missing teeth, it was not assessed. The second category of treatment needs (TN II) was recorded in 42 patients (41.18%). A similar result was obtained by Cotti et al., whose results showed TN II to be the case for 44.87% of the study group [30].

They should be instructed to maintain proper oral hygiene, perform supra- and under-gingival scaling, and remove overhanging fillings and crowns at the gingival margin. The third category of treatment needs (TN III) was determined in four people (3.92%) with gingival pockets deeper than 6 mm. TN III indicates severe generalized periodontitis and the need for comprehensive periodontal treatment in these patients. The assessment of the CPI with respect to the TN allows to establish a general plan of periodontal treatment in the examined patients, which can be started depending on the value of blood parameters.

A study carried out by Shankarapillai et al. in 73 adults with a diagnosis of AML reported that about 75% of them had either fair or poor oral hygiene, assessed using traditional clinical indices. A statistically significant association between dental plaque levels and both gingival overgrowth and periodontal index (*p* < 0.001) was observed. More than one-third of patients were reported to have significant or life-threatening infections, most of which were of bacterial origin [31]. There was only one comparable study in which the authors found that poor oral health did not affect the induction outcome in children with acute leukemia [32]. The parameter of great clinical importance is the occurrence of infectious complications during chemotherapy. The analysis model showed that 318 out of 1000 patients suffer from infectious complications, of which 68 infections may be caused by an oral foci [33].

In a study conducted by Laine et al. on 56 patients, it was stated that individuals with untreated periodontal inflammation receiving chemotherapy presented a higher prevalence of febrile episodes than those with a healthy periodontium [34].

Oral bacteremia in patients with leukemia undergoing chemotherapy is still discussed and some authors find it controversial [35,36].

In our study, no deterioration in the oral hygiene of patients treated for leukemia during one cycle of chemotherapy was found. This might be due to the generally poor periodontal status and oral hygiene of the patients examined before chemotherapy. Therefore, awareness of the importance of dental care along with the medical management of these patients should be spread, and the involvement of multi-disciplinary teams is recommended.

The limitations of the study were the conditions of the dental examination that was not performed in a dentist’s office. Additionally, the patients were people of poor general condition and with improper oral hygiene. The patient population treated in this hospital usually consists of people from the voivodeship and cannot be extrapolated to the whole country.

## 5. Conclusions

1. In the majority of patients, both before and after chemotherapy, improper oral hygiene and severe generalized periodontitis were confirmed;
2. The cycle of chemotherapy that was used did not correlate with the change of patients’ oral hygiene and periodontium state;
3. Unsatisfactory oral hygiene and periodontal health have to be addressed with urgent dental treatment to avoid systemic complications in leukemic patients.

## Figures and Tables

**Table 1:** Symptoms and categories of treatment needs according to CPITN.

Code of Disease Symptoms (CPI)		Category of Treatment Needs (TN)	
0	healthy periodontium	I	oral hygiene instruction
1	gums bleeding after gentle probing	I	oral hygiene instruction
2	supragingival and subgingival calculus, overhanging margins of fillings and crowns	II	oral hygiene instruction, supra- and subgingival scaling, removal of overhanging restorations and crowns
3	gingival pockets from 3.5 to 5.5 mm	II	oral hygiene instruction, supra- and subgingival scaling, removal of overhanging restorations and crowns
4	gingival pockets from 6 mm	III	oral hygiene instruction, supra- and subgingival scaling, removal of overhanging restorations and crowns, comprehensive treatment

**Table 2:** The type of leukemia in the study group.

	*n*	%
Chronic lymphocytic leukemia CLL	17	16.67
Chronic myeloid leukemia CML	10	9.80
Acute lymphoblastic leukemia ALL	16	15.69
Chronic hairy cell leukemia CHL	1	0.98
Acute myeloid leukemia AML	55	53.92
Acute promyelocytic leukemia APL	3	2.94
Total	102	100.00

**Table 3:** Chemotherapy cycle versus SBI index.

	*n*	Min	Max	M	SD	Me	*p*
Before treatment	87	23.00	100.00	63.83	24.76	59.00	0.5142
After treatment	87	23.00	100.00	65.25	24.54	65.00	

**Table 4:** Chemotherapy cycle versus API index.

	*n*	Min	Max	M	SD	Me	*p*
Before treatment	87	33.00	100.00	74.38	23.28	80.00	0.8816
After treatment	87	33.00	100.00	73.48	23.57	80.00	

**Table 5:** Chemotherapy cycle versus CPI.

	CPI									
	0		1		2		3		4	
	*n*	%	*n*	%	*n*	%	*n*	%	*n*	%
Before treatment	0	0	30	29.41	25	24.50	17	16.6	4	3.92
After treatment	0	0	30	29.41	25	24.50	17	16.6	4	3.92

**Table 6:** Treatment needs in examined patients.

	*n*	%
N/A—the index has not been marked	26	25.49
I	30	29.41
II	42	41.18
III	4	3.92
	102	100.00

**Table 7:** Patient age and the CPI determination.

	≤56		>56		*p*
	*n*	%	*n*	%	
No	8	14.81	18	37.50	<0.01
Yes	46	85.19	30	62.50	

**Table 8:** The categories of treatment needs and the age of patients.

	TN								*p*
	I		II		III		No		
	*n*	%	*n*	%	*n*	%	*n*	%	
≤56	22	73.33	22	52.38	2	50.00	8	30.77	0.0173
>56	8	26.67	20	47.62	2	50.00	18	69.23	

**Table 9:** The association between periodontal indices before (1) and after (2) chemotherapy versus type of leukemia (chronic and acute).

	Chronic			Acute			*p*
	*n*	M	SD	*n*	M	SD	
API (1)	28	70.96	22.71	59	76.00	23.56	0.3588
SBI (1)	28	62.50	25.06	59	64.46	24.81	0.6661
API (2)	28	67.21	22.20	59	76.46	23.80	0.0893
SBI (2)	28	60.86	21.10	59	67.34	25.92	0.2776

## Data Availability

Not applicable.
